# Sonication in the diagnosis of fracture-related infections (FRI)—a retrospective study on 230 retrieved implants

**DOI:** 10.1186/s13018-021-02460-z

**Published:** 2021-05-13

**Authors:** Petri Bellova, Veronika Knop-Hammad, Matthias Königshausen, Thomas A. Schildhauer, Jan Gessmann, Hinnerk Baecker

**Affiliations:** 1 Department of Orthopedics and Trauma Surgery, BG University Clinic Bergmannsheil Bochum, Bürkle-de-la-Camp-Platz 1, 44789 Bochum, Germany; 2 Department of Microbiology, BG University Clinic Bergmannsheil Bochum, Bürkle-de-la-Camp-Platz 1, 44789 Bochum, Germany

**Keywords:** Sonication, Fracture-related infection, Biofilm, Sensitivity, Specificity

## Abstract

**Background:**

In fracture-related infections (FRI), both the diagnosis of the infection and the identification of the causative pathogen are crucial to optimize treatment outcomes. Sonication has been successfully used for periprosthetic joint infections (PJI); however, its role in FRI remains unknown. Our aim was to determine the diagnostic accuracy (sensitivity, specificity) of sonicate fluid culture (SFC). The primary objective was to compare SFC with peri-implant tissue culture (PTC) overall and among subgroups using the consensus definition by Metsemakers et al. The secondary objective was to determine the yield of SFC in possible fracture-related infections (PFRI).

**Methods:**

From March 2017 to May 2019, 230 cases of retrieved implants were retrospectively reviewed. To perform sonication, explants were placed in sterile polypropylene jars intraoperatively. After treatment in an ultrasonic bath (Bandelin, Berlin, Germany), sonicate fluid was incubated into blood culture bottles, and conventional culturing was eventually performed. Sensitivity and specificity were determined using two-by-two contingency tables. McNemar’s test was used to compare proportions among paired samples while Fisher’s exact test was used for comparison between categorical variables.

**Results:**

Of the 230 cases, 107 were identified as FRI, whereas 123 were aseptic revision cases (ARC). Of the latter, 105 were labeled as PFRI. Sensitivity of SFC was higher in comparison with PTC, although this did not reach statistical significance (90.7% vs. 84.1%; *p* = .065). The specificity of SFC was significantly lower than that of PTC (73.2% vs. 88.6%; *p* = .003). In PFRI, SFC yielded significantly more positive results than PTC (33/105 vs. 14/105; *p* = .003). Overall, 142 pathogens were identified by SFC, whereas 131 pathogens were found by PTC.

**Conclusions:**

We found that sonication of fracture fixation devices may be a useful adjunct in FRI, especially in “low-grade” infections lacking confirmatory clinical criteria. Standardized diagnostic protocols are warranted in order to further optimize the diagnostic accuracy.

## Background

Fracture-related infections (FRI) pose a major clinical challenge and may have devastating consequences, including non-union, multiple revision surgeries, or even amputation. The infections are almost exclusively acquired exogenously [1]. While infection rates for closed fractures are estimated at approximately 1%, they can rise to up to 30% for Gustillo and Anderson type III open fractures due to the colonization during the trauma, disturbed wound healing, or late soft tissue coverages [2, 3].

Infections may be clinically apparent, but they may also be associated with subtle signs such as chronic pain, non-union or implant loosening in radiographs. Moreover, conventional tissue samples, which have been the gold standard thus far, are prone to high culture-negative rates of up to 42% [4, 5]. One of the reasons might be the biofilm formation which is associated with medical implants. In a mature biofilm, microorganisms are sessile, embedded in a polymeric matrix and have changed their phenotype, which makes their detection very difficult [6, 7]. The diagnosis of FRI is not always straightforward, and until recently, FRI has lacked a clear working definition. Recently, a consensus definition was implemented by an expert group comprising scientific and medical organizations with the support of the AO Foundation [8].

Sonication helps to dislodge biofilms from the implant surface and was popularized by Trampuz et al. [9]. Since then, it has been implemented routinely in an increasing number of clinics and has generally shown superior sensitivity when compared with tissue samples in a wide array of studies, most of which were recently summed up in two major meta-analyses [10, 11]. Most studies, however, have investigated prostheses and their components while the few studies that have focused on the sonication of fracture fixation devices have had small sample sizes and have lacked a clear working definition for FRI [12]. Therefore, there is a clear need for further research on this very topic.

Our aim was therefore to investigate the diagnostic accuracy of sonication in association with fracture fixation devices. The primary objective was to compare sonicate fluid culture (SFC) with peri-implant tissue culture (PTC) overall and among subgroups using the most recent consensus definition by Metsemakers et al. [8] as the reference standard. The secondary objective was to determine the yield of SFC in comparison with PTC in possible fracture-related infections (PFRI).

## Materials and methods

A multidisciplinary approach including the department of orthopedics and trauma surgery and the department of microbiology allowed for the conduction of this study.

**Fig. 1:**
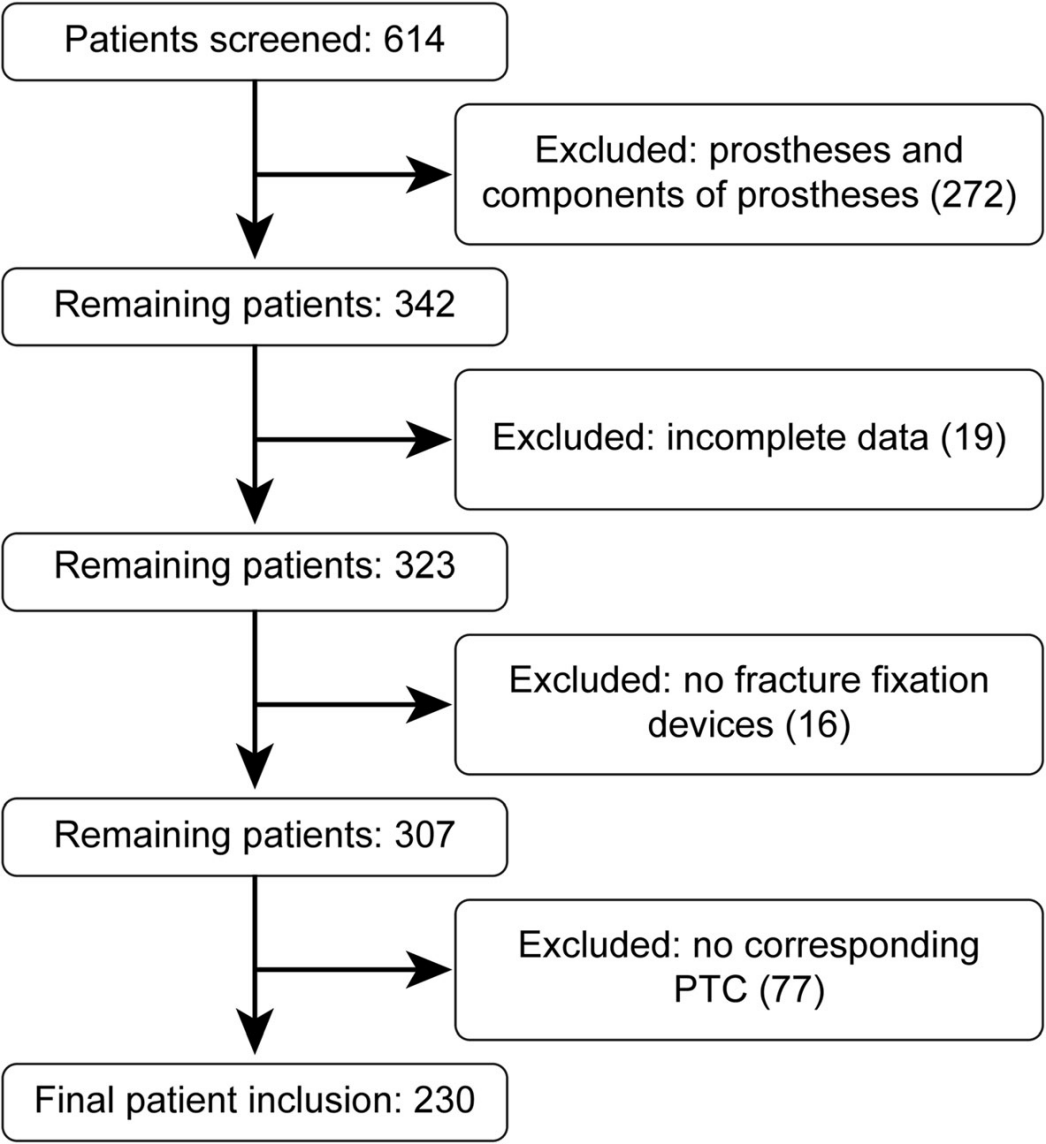
Flow chart of patient inclusion

Implants were submitted for sonication to our microbiological laboratory. From March 2017 to May 2019, 614 consecutive cases of retrieved implants were screened. Exclusion criteria were prostheses and their respective components (*n* = 272), cases with incomplete data (*n* = 19), retrieved materials other than classic orthopedic metalwork, such as fiber tapes, buttons or bioscrews (*n* = 16), and cases with no corresponding peri-implant tissue cultures (PTC) (*n* = 77). Finally, 230 cases were retrospectively included and reviewed (Fig. 1).

The approval of the local Ethical Review Committee was obtained (19-6603).

### Definition

**Fig. 2:**
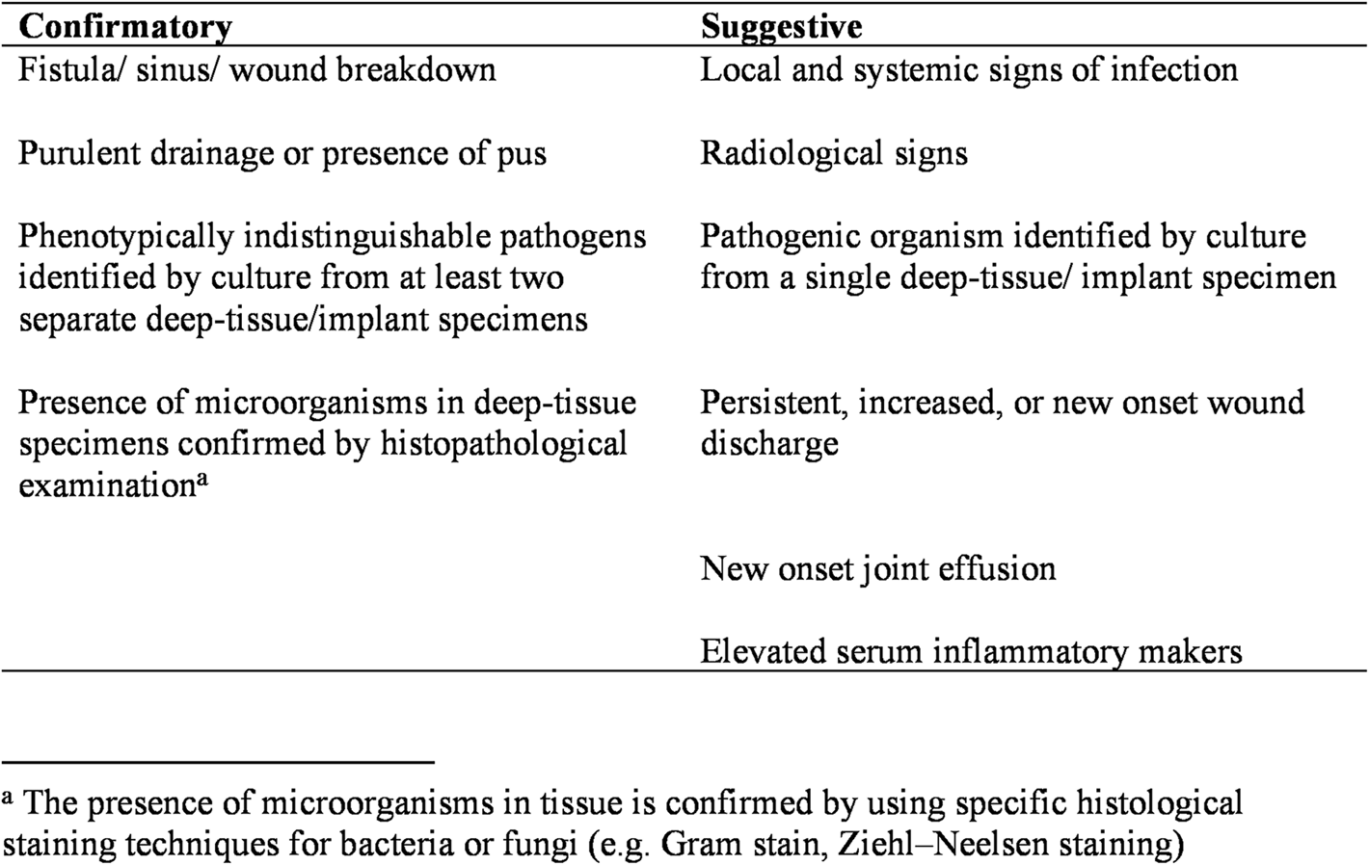
FRI definition by Metsemakers et al. [8]: the presence of one or more confirmatory criteria defines FRI. Suggestive criteria might hint at FRI but are not sufficient for definition

FRI was defined according to the consensus definition by Metsemakers et al. [8] (Fig. 2). A case was defined as infected if at least one confirmatory criterion was met. If only suggestive or no criteria were met, we defined the case as an aseptic revision case (ARC). Among these, we analyzed those with at least one suggestive criterion of infection in a separate analysis. These cases were labeled PFRI. Baseline characteristics were compared for all groups (age, sex, age of implants, number of PTC taken).

### Subgroups

The two groups, “FRI” and “ARC,” were subdivided into subgroups regarding diagnosis, affected bone, fracture fixation device, open fracture, early vs. delayed vs. late infection, and previous antimicrobial treatment. Furthermore, we conducted a subgroup analysis excluding all cases with a sinus and/or wound breakdown, a subgroup analysis excluding all cases with less than three corresponding tissue cultures, as well as a subgroup analysis excluding both.

Early infection was defined as an onset of symptoms within 2 weeks after the implantation of the device, delayed infections incorporated a range between 2 and 10 weeks, whereas late infections occurred more than 10 weeks after the implantation [1]. Previous antibiotic treatment was defined as any administration of antibiotics within 14 days before surgery.

### PTC

Intraoperative sampling of PTC incorporated the retrieval of deep tissue samples from the macroscopically most suspicious area of infection, preferably in the vicinity of the implant. During retrieval, contact with the skin was avoided to minimize cross-contamination. Samples from sinus tracts were avoided. Following removal, each device was placed immediately into a sterile, single-use, airtight container. In the microbiology laboratory, samples were prepared using forceps and scalpels under laminar air flow. Aliquots of the tissue were subsequently placed on different aerobic and anaerobic culture plates and growth media (blood agar, chocolate agar, Schaedler agar, brain-heart infusion, Wilkens–Chalgren infusion). Culture was performed under human body temperature conditions (37 °C) for 14 days. Isolates were identified by matrix-assisted laser desorption ionization-time of flight (MALDI-TOF) mass spectrometry (Bruker, UK Ltd.). Drug susceptibility testing was performed using manual EUCAST methods. In keeping with existing guidelines, two or more samples positive with an indistinguishable organism were used to define a microbiological positive while one pathogenic organism in one deep sample was a suggestive criterion for infection.

### SFC

In the operating room, explants were placed in sterile polypropylene containers that were opened immediately prior to component explantation. In the laboratory, explants were immersed in Ampuwa® (Fresenius Kabi Deutschland GmbH; Bad Homburg, Germany) solution, vortexed for 30 s, and treated in an ultrasonic bath (BactoSonic; Bandelin, Berlin, Germany) for 60 s at 80% *p* = 160 W, followed by another 30 s of vortexing. Subsequently, 10 ml of sonicate fluid were inoculated into BacTec Plus Aerobic/F and BacTec Lyte/10 Anaerobic/F bottles (BD Diagnostics, Sparks, MD), respectively. BacTec bottles were incubated at 37 °C for 10 day or until they flagged positive. Gram stain was performed on all isolates. Positive Bactec bottles were subcultured onto agar. Culture and identification of organisms was performed as described above.

### Statistical analysis

This study was a retrospective comparative study on the diagnostic value of sonication. Baseline characteristics of infected and noninfected cases were summarized as frequencies and percentages or means with standard deviation and the range in parentheses. The sample size of this study was computed with a .95 confidence interval using a broadly accepted method by Krummenauer et al [13]. The diagnostic accuracy (sensitivity, specificity) of SFC and PTC were calculated using two-by-two contingency tables. McNemar’s chi-squared test of paired proportion [14] was used to compare the sensitivity and specificity of the different culture methods. In order to compare the yield of SFC and PTC in PFRI, Fisher’s exact test [15] was used. Differences were considered significant when the *p* value was <.05 (two-tailed).

The detection of microorganisms in SFC and PTC was recorded. The pathogens were summarized in the groups depicted in Table 1. Statistical analysis was performed using SPSS software (V.27, IBM Corporation; Armonk, NY, USA).

**Table 1:** Groups of microorganisms and their respective prevalences. FRI, fracture-related infection; PFRI, possible fracture-related infection; PTC, peri-implant tissue culture; SFC, sonicate fluid culture

Group		Prevalence					
		FRI		PFRI		Overall	
		SFC	PTC	SFC	PTC	SFC	PTC
CoNS	Coagulase-negative staphylococci	24	27	18	3	42	30
MSSA	Methicillin-susceptible *S*. *aureus*	34	29	1	0	35	29
MRSA	Methicillin-resistant *S*. *aureus*	5	5	0	1	5	6
GNB	Gram-negative bacilli^a^	24	25	3	1	27	26
ENT	Enterococci	7	8	0	2	7	10
STR	Streptococci	3	4	1	1	4	5
ANAER	Anaerobes^b^	3	5	5	3	8	8
OTH	Other microorganisms^c^	7	10	7	7	14	17
**Total**		**107**	**113**	**35**	**18**	**142**	**131**

^a^Including *E*. *coli*, *Pseudomonas aeruginosa*, *Klebsiella pneumoniae*, *Enterobacter cloacae complex*, *Serratia marcescens*, *Proteus spp*. (=species), *Citrobacter spp*.

^b^Including *Cutibacterium spp*. and *Finegoldia magna*

^c^Including Fungi, *Corynebact*. *spp*., and others

## Results

A total of 230 implant removals for any cause were included in our study. The baseline characteristics are shown in Table 2. A total of 107 implants met the definition criteria for an infection, whereas 123 were defined as ARC. Among these, 105 met at least one suggestive criterion. These PFRI were analyzed separately. Eight different subgroup analyses were conducted which are depicted in Table 3.

**Table 2:** Baseline characteristics overall of FRI and of ARC. ARC, aseptic revision case; FRI, fracture-related infection

	Overall		FRI (***n*** = 107)		ARC (***n*** = 123)	
Age, years *(median*, *[range])*	61	*6-99*	58	*8–93*	63	*6–99*
Sex, male *(no*., *[%])*	119	*51*.*7*	61	*57*.*0%*	58	*47*.*1*
Age of implants, months *(median*, *mean*, *[range])*	4; 19.9	*0–540*	6; 25.2	*0–540*	4; 15.3	*0–348*
Number of samples taken *(mean*, *`range])*	2.45	*1–8*	2.51	*1–8*	2.40	*1–8*

**Table 3:** Different combinations of SFC and PTC. ARC, aseptic revision case; FRI, fracture-related infection; PFRI, possible fracture-related infection; PTC, peri-implant tissue culture; SFC, sonicate fluid culture

	Overall	FRI	ARC	PFRI
**SFC (+) PTC (-)**	38	9	29	29
**SFC (+) PTC (+) C**^**a**^	63	63	0	
**SFC (+) PTC (+) D**^**b**^**, add**^**c**^ **SFC**	6	6	0	
**SFC (+) PTC (+) D, add PTC**	11	11	0	
**SFC (+) PTC (+) D, diff**^**d**^	12	8	4	4
**SFC (-) PTC (-)**	88	8	80	62
**SFC (-) PTC (+)**	12	2	10	10

^a^C, concordant

^b^D, discordant

^c^Add, additional pathogen detected by (either SFC or PTC)

^d^Diff, different pathogens

Overall, SFC was positive 130 times, whereas PTC was positive 104 times. Among 107 defined FRIs, 97 were correctly identified by SFC (sensitivity 90.7%), while 90 were correctly identified by PTC (sensitivity 84.1%). This difference did not reach statistical significance (*p* = .065). Out of 123 ARC, 90 were accurately identified as negative by SFC (73.2%), whereas 109 were accurately negative by PTC (88.6%). This difference regarding specificity was statistically significant (*p* = .003).

Among the 105 PFRI, 43 had elevated inflammatory markers, 15 had local or systemic infectious signs, 89 had radiological signs, 5 had a persistent/ increased or new onset wound discharge, and 8 had a new onset joint effusion while 43 had a pathogen identified by a single deep tissue culture or sonication specimen. SFC was positive in 33 cases, whereas PTC was positive in 14 cases. This difference was statistically significant (*p* = .003).

The subgroup analyses are shown in Table 4. Sensitivity was equal throughout all investigated subgroups, whereas specificity was significantly higher for PTC in cases involving the femur (*p* = .013), in cases involving plates and screws (*p* = .004), in delayed infections (*p* = .004), when preoperative antibiotics were not administered (*p* = .001) as well as when infections involving a sinus tract were excluded (*p* = .003).

**Table 4:** Subgroup analysis and respective results for sensitivity and specificity. PTC, peri-implant tissue culture; SFC, sonicate fluid culture

	Total (%)		Sensitivity			Specificity		
			SFC	PTC	*p*	SFC	PTC	*p*
**Initial diagnosis**								
Suspected infection (local signs)	93	40.4	89.2	84.3	.22	50.0	30.0	.63
Non-union	68	29.6	92.3	76.9	.63	78.2	90.9	.12
Implant dislocation	47	20.4	100.0	90.9	–	80.6	88.9	.51
Peri-implant fracture	8	3.5	–^a^	–	–	50.0	87.5	.38
Hardware irritation	6	2.6	–	–	–	50.0	83.3	.63
Chronic osteitis	5	2.2	–	–	–	60.0	100.0	–
Posttraumatic osteoarthritis	3	1.3	–	–	–	100.0	100.0	–
**Bone**								
Femur	93	40.4	93.1	89.7	1	76.6	93.8	**.013**
Lower leg	71	30.9	90.4	82.7	.13	73.7	78.9	1
Humerus and shoulder girdle	29	12.6	100.0	87.5	–	66.7	90.5	.13
Spine	15	6.5	91.7	83.3	1	33.3	66.7	1
Forearm	9	3.9	100.0	100.0	–	87.5	100.0	–
Pelvis	8	3.5	100.0	100.0	–	60.0	60.0	1
Foot	5	2.2	0.0	0.0	–	66.7	66.7	1
**Fracture fixation device**								
Plate and screws	167	72.6	90.0	82.9	.18	73.2	89.7	**.004**
Nail	26	11.3	100.0	93.8	–	60.0	100.0	–
Screws	24	10.4	83.3	77.8	1	83.3	66.7	1
Multiple	9	3.9	100.0	100.0	–	82.2	71.4	1
(Cerclage) wires	4	1.7	100.0	100.0	–	66.7	100.0	–
**Early vs. delayed vs. late**^b^								
Early	11	4.8	100.0	100.0	–	77.8	77.8	1
Delayed	71	30.9	86.4	77.3	.22	59.3	92.6	**.004**
Late	148	64.3	93.4	88.5	.38	77.0	88.5	.08
**Preoperative antibiotics**^c^								
Yes	35	15.2	85.2	70.4	.22	62.5	50.0	1
No	195	84.8	92.5	88.8	.38	73.9	91.3	**.001**
**Sinus excluded**^d^	**180**	78.3	98.2	93.0	.25	73.2	88.6	**.003**
**3+ tissue cultures**^e^	**88**	38.3	86.0	79.1	.45	75.6	82.2	.61
**Sinus excluded AND 3+ tissue cultures**	**66**	28.7	100	90.5	–	75.6	82.2	.61

^a^–, missing values/not calculable

^b^Early: < 2 weeks after device implantation; delayed: 2–10 weeks; late: > 10 weeks

^c^Any administration of antibiotics within 14 days prior to surgery

^d^Patients with sinus tract and/or wound breakdown excluded from sub-analysis

^e^Patients with less than three retrieved corresponding tissue cultures excluded from sub-analysis

Including polymicrobial cases, 142 pathogens were detected through SFC, while 131 were detected through PTC in all 230 cases reviewed. In FRI alone, 107 and 113 pathogens were detected through SFC and PTC, respectively, whereas in possibly infected cases, 35 and 18 cases were detected through SFC and PTC, respectively.

The relative distribution of pathogens according to their respective groups is shown in Table 5. While the most frequently identified microorganisms in FRI were *S*. *aureus*, coagulase-negative staphylococci (CoNS) were the most frequently detected group in PFRI.

**Table 5:** Groups of pathogens and their respective distribution overall, in FRI and in PFRI. ANAER, anaerobes; CoNS, coagulase-negative staphylococci; ENT, enterococci; FRI, fracture-related infection; GNB, gram-negative bacilli; MSSA, methicillin-sensible *S*. *aureus*; MRSA, methicillin-resistant *S*. *aureus*; OTH, other (microorganisms); PFRI, possible fracture-related infection; PTC, peri-implant tissue culture; SFC, sonicate fluid culture; STR, streptococci

Pathogen	Overall (***n*** = 230)				FRI (***n*** = 107)				PFRI (***n*** = 105)			
	SFC	(%)	PTC	(%)	SFC	(%)	PTC	(%)	SFC	(%)	PTC	(%)
NEG^a^	100	43.5	126	54.8	10	9.3	17	15.9	72	68.6	91	86.7
CoNS	35	15.2	17	7.4	19	17.8	16	15.0	16	15.2	1	1.0
MSSA	31	13.5	26	11.3	30	28.0	26	24.3	1	1.0	0	.0
MRSA	5	2.2	5	2.2	5	4.7	4	3.7	0	.0	1	1.0
GNB	19	8.3	15	6.5	16	15.0	14	13.1	3	2.9	1	1.0
ENT	5	2.2	3	1.3	5	4.7	2	1.9	0	.0	1	1.0
STR	3	1.3	3	1.3	3	2.8	3	2.8	0	.0	0	.0
ANAER	8	3.5	5	2.2	3	2.8	4	3.7	5	4.8	1	1.0
OTH	10	4.3	6	2.6	4	3.7	3	2.8	6	5.7	3	2.9
POLY^b^	14	6.1	24	10.4	12	11.2	18	16.8	2	1.9	6	5.7
	**230**		**230**		**107**		**107**		**105**		**105**	

^a^NEG, culture negative

^b^POLY, polymicrobial = detection of two or more pathogens

## Discussion

FRI remain a serious complication in orthopedic trauma care [16]. Current success rates following infection only range between 70 and 90% [17, 18]. Optimization of diagnostic and treatment concepts is therefore crucial.

In terms of diagnosis, sonication may provide an additional benefit to tissue samples. Sonication helps dislodge biofilm bacteria from implant surfaces. While the original method described by Trampuz et al. [9] has generally remained unchanged, minor modifications, such as the incubation of sonicate fluid in blood culture bottles, have helped improve detection rates [19, 20]. The latter incubation method was used in this study.

Overall, in the majority of studies investigating PJI, sonication has shown an improved sensitivity while displaying equal specificity when compared with conventional tissue cultures [10, 11]. However, studies on the sonication of fracture fixation devices have been scarce [12] and difficult to compare due to different study conditions. One of the few studies focusing solely on fracture fixation devices was conducted by Yano et al. [21]. In this study, the authors presented the results of 180 osteosyntheses and found a significantly higher sensitivity for sonicate fluid culture when compared with tissue cultures. One of the drawbacks, which was also recognized by the authors, was the lack of a custom definition for infection which was not developed until recently [8, 22]. In most other studies, fracture fixation devices were pooled together with prostheses, which makes it impossible to determine their diagnostic performance separately [23–26]. The study by Dudareva et al. [27], meanwhile, while also investigating both, prostheses and fracture fixation devices, conducted a sub-analysis on the results of 111 orthopedic fixation devices. Contrary to most other studies on this topic, the authors found a higher sensitivity for tissue samples when compared with sonication using a clinical definition for infection (64% vs. 45%; *p* = .002). The authors concluded that if performed correctly (sufficient number of samples, etc.), tissue cultures are more sensitive than sonication and underscored the role of sonication as an additive tool but not as a substitute for tissue cultures. Simultaneously, the authors pointed out the different methodology used by different studies, such as insufficient tissue sampling, which has led to variable results [27].

When adopting the new definition criteria [8], we found a trend towards improved sensitivity for sonication when compared with tissue samples. The specificity, meanwhile, was superior for tissue samples.

Since it is assumed that a sinus tract influences the detection rate of both sonication and tissue samples due to continuous rinsing of the infected fluid out of the wound, we conducted a subgroup analysis excluding all cases with a draining sinus. Although both SFC and PTC had a high sensitivity under this scenario, there was no difference between the two.

Also, the fact that a previous antibiotic therapy did not give sonication an advantage regarding sensitivity was contrary to the findings of most studies on PJI in which this sub-analysis was conducted [21, 25, 26]. In addition, we expected sonication to provide an advantage in later manifestations of symptoms when a biofilm had already formed on implant surfaces [9]. However, contrary to the study by Puig-Verdi et al. [26], sensitivity did not differ regardless of early, delayed, or late manifestation of symptoms in our study. The lacking statistical significance of these sub-analyses may also be due to the rather small sample sizes of the respective subgroups.

The respective incidences of identified microorganisms found in our study were in concordance with those found by Yano et al. [21]. The high detection rates for gram-negative bacilli are a reflection of the increasing incidence of gram-negative infections [28, 29]. The sonication-based detection rate in aseptic or presumably aseptic cases was also in concordance with comparable studies [30, 31]. In PFRI, CoNS combined for more than half of all detected pathogens when also including polymicrobial cases. This was also in concordance with comparable studies [30, 32]. As already known from previous studies, sonication is more susceptible to detect low-virulence pathogens [33, 34]. In addition to CoNS, the role of anaerobes has often been emphasized. In our study, the rate of anaerobes found was lower than in one recent study on noninfected fracture fixation devices [35].

Our study has several drawbacks. As the establishment of sonication in the diagnosis of FRI was new in our clinic, diagnostic and treatment protocols were not yet standardized. This included a failure to determine CFUs in the process of sonication. According to Trampuz et al. [9], the threshold for sonicate fluid cultures to be considered an infection is 50 CFU/ml, whereas lower thresholds may be indicative of an infection when the patient is under antibiotic treatment or when pathogens of high virulence are detected [36]. After incubation of sonicate fluid in blood culture bottles, however, CFU counting is no longer valid and therefore needs to be done before the incubation. Generally, the counting of CFUs would be a useful tool in distinguishing between contamination and infection, thus improving specificity.

Although considered, the role of sonication is not explicitly highlighted within the definition criteria Metsemakers et al. [8] which are very straightforward. Thus, it is unclear how to incorporate sonication results with an arbitrary number of CFU/ml into the definition criteria, since a single implant specimen with > 50 CFU/ml would not suffice in order to secure the diagnosis under the current criteria.

A further drawback is the lack of standardized tissue sampling. Unfortunately, in only 38.3% of cases a sufficient number of tissue samples were retrieved. Within this subgroup, the diagnostic accuracy of sonication and tissue samples were equal. Our results show that the performance of sonication and tissue cultures might be similar- provided that a sufficient number of tissue samples is retrieved [22]. If sonication is not available at all, 5 or more samples might be needed in order to achieve comparable diagnostic value [9, 22].

As suggested by the new definition, cases fulfilling at least one suggestive criterion can be labeled as PFRI. Within these, sonication flagged positive more often than tissue samples. Depending on the clinical situation, the label PFRI has to be taken seriously, for example, in cases where confirmatory infection might manifest itself at some point in the future. In the future, the suggestive criteria need to be further validated, and a scoring system comparable to the new PJI definition [37] might be established. Due to the retrospective nature of this study, these cases were not followed up. We acknowledge that this is a major drawback of the study as it is difficult to assess which percentage of PFRI was actually infected in the long term. A further drawback is the lack of standardized histological sampling. Although histological sampling was performed in a total 56 cases and signs of infections were described in 29, there was no standardized assessment. Thus, histology had to be excluded from consideration, which might have led to some missed infections. The inclusion of quantitative neutrophil analysis with histological evaluation, especially in chronic/late onset cases, has been recently proposed [22, 38].

## Conclusion

Sonication was shown to be a useful adjunct in the diagnosis of FRI displaying a high sensitivity. Knowing this, it should not only be routinely used in the management of PJI, but also of FRI. While a sufficient number of tissue samples may compensate for a lack of sonication, the latter can be especially useful in clinically inapparent or possible infections. Standardized diagnostic protocols, including the routine implementation of CFU, higher sample sizes, as well as follow-up of patients with unconfirmed infections, are important points to be addressed in future studies.

## Data Availability

The datasets used and/or analyzed during the current study are available from the corresponding author on reasonable request.

## Abbreviations

ARC: Aseptic revision case
CFU: Colony-forming units
CoNS: Coagulase-negative staphylococci
FRI: Fracture-related infection
PFRI: Possible fracture-related infection
PJI: Periprosthetic joint infection
PTC: Peri-implant tissue culture
SFC: Sonicate fluid culture
